# The impact of reverse abdominal breathing on lower limb muscle strength and muscle synergy characteristics in Tai Chi Chuan

**DOI:** 10.3389/fbioe.2025.1579139

**Published:** 2025-06-27

**Authors:** Yanyu Yin, Yanlong Zhang, Minghui Wang, Yuhang Zhu, Gengchao Bi, Shuo Zhang

**Affiliations:** ^1^ College of Sports and Health Sciences, Mudanjiang Normal University, Mudanjiang, China; ^2^ Graduate School, Harbin Sport University, Harbin, China; ^3^ DUT-BSU Joint Institute, Dalian University of Technology, Dalian, China

**Keywords:** Tai Chi Chuan, reverse abdominal breathing, muscle activation, muscle synergy, posture control

## Abstract

**Objective:**

To investigate the impact of two different breathing methods on lower limb muscle strength and muscle synergy in Tai Chi Chuan, and to reveal the joint control strategies of posture stability and synergy associated with the reverse abdominal breathing in Tai Chi Chuan.

**Methods:**

Fifteen subjects aged between 33 and 43 years with over 5 years of Tai Chi Chuan experience were selected. High-speed motion capture, a three-dimensional force platform, and surface electromyography were used to collect kinematic and kinetic parameters of Tai Chi Chuan movements under natural breathing and reverse abdominal breathing conditions. Lower limb muscle dynamics were analyzed through computational modeling, and neuromuscular coordination patterns were investigated using muscle synergy analysis techniques. Paired-sample t-tests were used to analyze the differences in various parameters between the two breathing conditions, with significance at P < 0.05.

**Results:**

The peak muscle strength of the gluteus maximus, rectus femoris, and biceps femoris was consistently greater during reverse abdominal versus natural breathing (P < 0.05), as was tibialis anterior co-activation (P < 0.05). Conversely, lateral gastrocnemius-tibialis anterior co-activation patterns showed distinctly lower values (P < 0.05). The stiffness of the knee and ankle joints significantly increased (P < 0.05). In terms of muscle synergy, in the synergy units dominated by the gluteus maximus, rectus femoris, and semitendinosus, the relative weight during reverse abdominal breathing was significantly higher than that during natural breathing (P < 0.05), exhibiting a more efficient muscle synergy.

**Conclusion:**

Reverse abdominal breathing in Tai Chi Chuan enhances muscle activation and synergy, which facilitates increased recruitment of lower limb muscles and optimize joint strategies, thereby improving the stability and coordination of posture control. Reverse abdominal breathing optimized the activation pattern of lower limb muscles, playing an important role in enhancing stability and coordination in individuals with over 5 years of Tai Chi Chuan experience, particularly in the contexts of physical fitness and sports rehabilitation.

## 1 Introduction

Breathing regulation, as an economical, convenient, and simple intervention method, has been applied in sports fitness and clinical rehabilitation (Zaccaro et al., 2018; He and Ren, 2025). In practical applications, the trunk muscle activity induced by breathing affects hip, knee, and ankle joint angles, thereby modulating lower limb muscle activation and postural coordination (Foskolou et al., 2022). This reflects the extent to which breathing, over time, influences posture stability, demonstrating a “breathing-posture” synchronization. In recent years, breathing regulation has gradually been recognized and validated. Previous studies have shown that breathing regulation plays an extremely important role in physical exercise (Kocjan et al., 2017). Breathing has a significant impact on trunk stability and postural stability during movement (Sengupta, 2012). It is crucial for the recovery of core muscle perturbations and neuromuscular coordination of the lower limbs.

Tai Chi Chuan (TCC) movements are slow and integrate awareness, action, and deep breathing, emphasizing the involvement of most muscle tissues in breathing coordination. Through a series of breathing exercises, TCC coordinates posture control and calms both body and mind (Wang et al., 2020), following the principles of “inhale as you rise, exhale as you fall” and “inhale to open, exhale to close.” Studies have found that long-term practice of TCC can effectively improve the physical stability of the elderly, enhancing their lower limb muscle strength, coordination, flexibility, proprioception, and other abilities (Min and Youn, 2022; Law and Li, 2014; Bai et al., 2023; Huang et al., 2015). The natural breathing in TCC primarily involves mixed-type breathing, where practitioners do not need to consciously control the depth, speed, or rhythm of their breath. In contrast, during reverse abdominal breathing, the abdomen actively contracts inward during inhalation, and expands outward during exhalation. This breathing mechanism regulates the stable variation of posture over time. In TCC practice, not only is deep breathing required, but more importantly, reverse abdominal breathing is emphasized. This reflects that breathing is a key component of mind-body practice. By integrating various sensory information, it coordinates neuromuscular activity control strategies, demonstrating the sensory system, motor loops, and muscle coordination to maintain posture stability (Kavounoudias and Roll, 2001). Although different breathing methods can activate muscles involved in breathing to some extent, stimulating trunk and core muscle strength, the unique breathing characteristics of TCC may influence muscle activity in other parts of the body. Therefore, the coordination of different breathing methods with posture in TCC may have a significant impact on posture control and the fitness benefits of practice.

Muscle synergy is the smallest unit of the central nervous system (CNS) controlling the musculoskeletal system to perform various movements (Stephens et al., 2017). The CNS coordinates different muscle synergies to maintain the body’s center of mass within a certain spatial range, thereby controlling posture stability and reducing body sway. By extracting muscle synergy effects, it is possible to identify specific forms of feedforward control incorporating electromyographic (EMG) signal information. Since the breathing process itself is an endogenous source of postural stability disturbance (Lespert et al., 2023), and muscle synergy control plays a crucial role in posture control, it is closely linked to breathing regulation. Therefore, muscle synergy theory can effectively explain the CNS’s control mechanisms over muscles, further revealing the intrinsic functional patterns of the neural system coordinating respiratory muscles.

The human standing posture is commonly modeled as a combination of a single and double inverted pendulum, with the inverted pendulum rotating around the ankle joint referred to as the “ankle strategy (Macpherson et al., 1989).” As the amplitude of postural sway increases, the contribution of the hip joint movement also increases, represented by the “hip strategy.” Compared to the ankle strategy, the hip strategy is primarily driven by core strength, which enhances the stability of the body. As TCC places high demands on lower limb muscle strength, postural control, and muscle synergy, the transition of TCC breathing training modes depends on the degree of stability and the motor strategies pre-selected based on the task, which aids in understanding the choice of strategies involving the hip, knee, and ankle joints. Currently, research on TCC, both theoretically and practically, mostly focuses on dynamic balance control and endurance performance (Shi et al., 2024). However, previous studies have not clearly addressed the effects of breathing regulation on neuromuscular control and joint strategy selection. The impact of breathing training on muscle strength, joint stiffness, muscle synergy, and activation remains unclear. There is a need to explore how reverse abdominal breathing enhances the CNS’s influence over muscle synergy coordination. Thus, we hypothesize that TCC reverse abdominal breathing will influence postural control, increase lower limb joint stiffness, improve both static and dynamic balance abilities, and enhance the activation and coordinated activation of lower limb muscles. These findings will provide preliminary evidence for integrating reverse abdominal breathing into tailored rehabilitation protocols (e.g., balance recovery in elderly populations) and sport-specific training regimes (e.g., martial arts athletes requiring refined postural control).

## 2 Materials and methods

### 2.1 Research subjects

**FIGURE 1:**
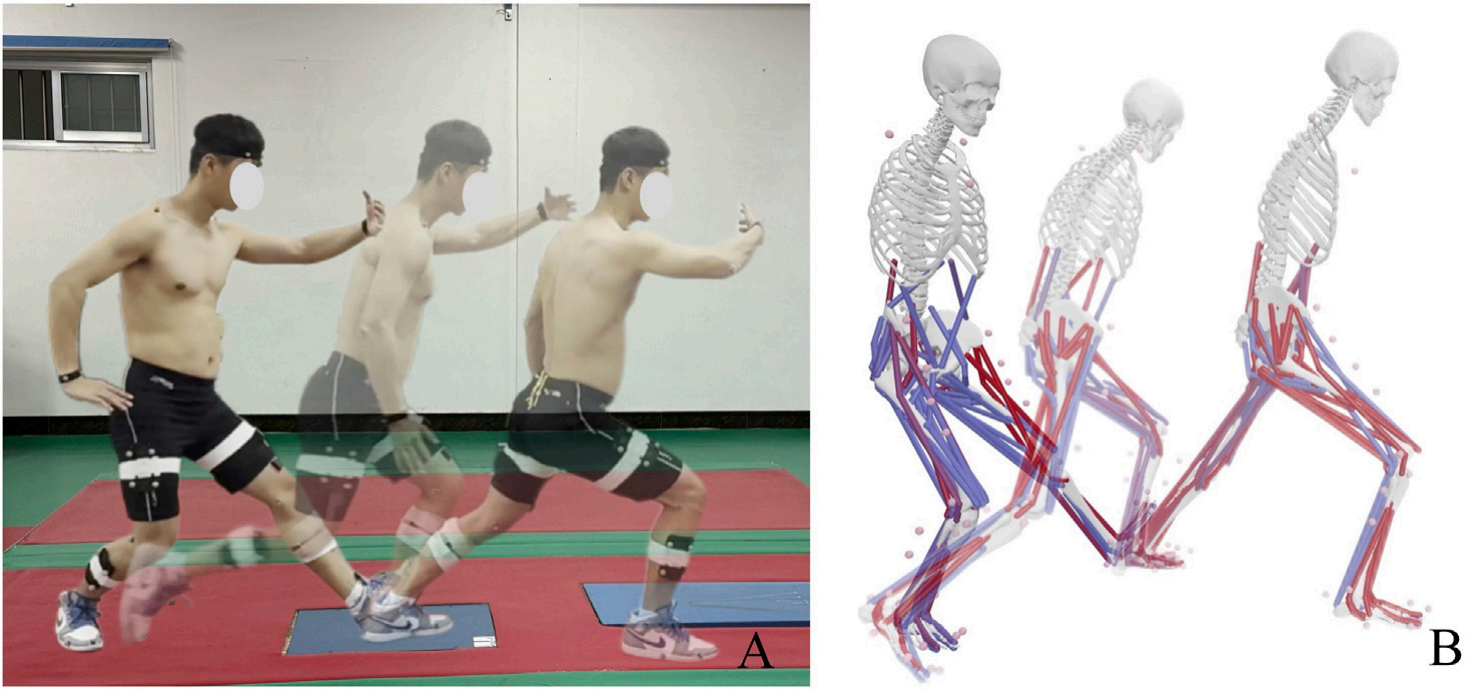
TCC movement and simulated by OpenSim. **(A)** Movement Process Schematic; **(B)** Movement Process Simulation Schematic. Blue represents inactive muscles, while red indicates activated muscles.

This study investigates the effects of natural breathing and reverse abdominal breathing on lower limb muscle activation during the typical TCC movement “Wild horse parts its mane (left side).” As a typical TCC movement, “Wild horse parts its mane” includes both a single-leg support phase and a swinging phase, which is similar to normal walking (Wu and Millon, 2008). The research focuses on the phase from the external rotation of the left foot to the forward shifting of the center of mass and the landing of the right foot, as illustrated in Figure 1A, analyzing the lower limb muscle characteristics during the left-leg support phase. The sample size was calculated using G*Power 3.1, with α = 0.05 and Power = 0.8, yielding a minimum required sample size of 15. Fifteen TCC practitioners (9 males, 6 females) with more than 5 years of practice experience were selected as participants (age: 38 ± 5.1 years, height: 1.74 ± 0.15 m, weight: 70.2 ± 7.5 kg). Participants were required to have no joint or muscle-related diseases, no health issues, and be able to complete the tests according to the standard procedure. Before the experiment, participants were thoroughly informed about the test movements and requirements, and written informed consent was obtained. This study was approved by the Ethics Committee of Mudanjiang Normal University, with approval number 2024004.

### 2.2 Testing protocol

The study utilized a 16-channel wireless telemetry surface EMG system, Trignolab version, produced by Delsys (United Stated), with a sampling frequency of 2,000 Hz. The system recorded the EMG activity of nine muscles in the participant’s left leg, including: gluteus maximus (G-max), long head of the biceps femoris (BF), rectus femoris (RF), semitendinosus (SM), vastus medialis (VM), vastus lateralis (VL), gastrocnemius medial (GM), gastrocnemius lateral (GL), and tibialis anterior (TA). Surface EMG sensors were attached to the most prominent areas along the muscle fibers of these nine muscles. The attachment locations followed the guidelines recommended by the International Society of Electrophysiology and Kinesiology (Barero et al., 2012). Before placement, the target muscle areas were shaved to remove surface hair, and the skin was cleaned with 75% alcohol to eliminate sweat and oils that could increase resistance. Ground reaction forces during the support phase of the participant’s left leg were measured using the AMTI BP400600 three-dimensional force platform produced by AMTI (United Stated), with a sampling frequency of 1,200 Hz. Three-dimensional motion capture was performed using the Qualisys high-speed motion capture system from Qualisys (Sweden), which included eight high-speed infrared cameras, with a sampling frequency of 200 Hz. Reflective markers were attached to the participant’s body based on the marker set of the OpenSim Gait2392_Simbody model to track their motion. Real-time data synchronization for the motion trajectories, force platform, and EMG signals was achieved using the Qualisys Track Manager 2021 software.

First, participants were instructed on the specific requirements of the test movements for both breathing methods. During natural breathing, the participant began from a posture with both hands holding the ball, the right leg supporting, and the left foot brought to the inside of the right foot with the toes touching the ground. The participant then rotated the torso to the left, stepping forward into a lunge while simultaneously separating the hands. The center of mass shifted backward, with the left foot externally rotated by 15°, and the right foot brought to the inside of the left foot with the toes touching the ground, all while holding the ball with both hands. The left foot became the support leg, and the participant then rotated the torso to the right, stepping forward into another lunge while separating the hands again. During this entire process, the breathing was not consciously controlled but followed naturally with the movement. For reverse abdominal breathing, the participant followed the same set of movements but synchronized breathing with the actions. Specifically, inhalation occurred during torso rotation and leg lifting, and the abdomen should actively contract. Exhalation took place during leg landing and the shifting of the center of mass forward, with the abdomen relaxing accordingly. Throughout the movement, there was a noticeable rise and fall of the abdomen. A 10-min stretching warm-up was conducted before performing the corresponding “Wild horse parts its mane” movements for data collection, and participants completed a 10-min reverse abdominal breathing training. They practiced synchronizing breath cycles with movements using a metronome set for 2-s inhalations during torso rotation/leg lift and 3-s exhalations during landing/weight shift, with verbal cues to actively contract the abdomen during inhalation. During trials, participants independently replicated this pattern while researchers visually verified abdominal rise/fall. Each breathing method was repeated three times, with a 30-s interval between each repetition of the same method and a 5-min interval between different methods.

### 2.3 OpenSim simulation process

The C3D file was exported from Qualisys Track Manager 2021 software and input into MATLAB R2021a. The c3dExport.m script in OpenSim was then run to perform axis conversion and generate the marker trajectory files and ground reaction force files required for the OpenSim simulation. For the modeling and simulation process, the Gait2392_Simbody musculoskeletal model was used in OpenSim to create a static model (Hua et al., 2024), and the model was scaled to match the body dimensions of the participant. Next, inverse kinematics and inverse dynamics calculations were performed to obtain kinematic and dynamic data such as muscle forces, joint angles, and joint torques. The measurement noise and modeling errors are reduced through smoothing of the motion trajectory and residual reduction algorithms. Joint stiffness was calculated using joint angles and joint torques, as shown in Equation 1. Finally, static optimization was used to compute muscle activation levels and force magnitudes. The activated portions of the muscles were shown in red, with deeper red indicating higher activation levels, as illustrated in Figure 1B. Muscle forces for muscles including G-max, BF, RF, SM, VM, VL, GM, GL, and TA were extracted.

$${Stiffness}_{joint}=∆{Torque}_{joint}/∆{Angle}_{joint}$$ (1)

### 2.4 EMG Signal Processing

**FIGURE 2:**
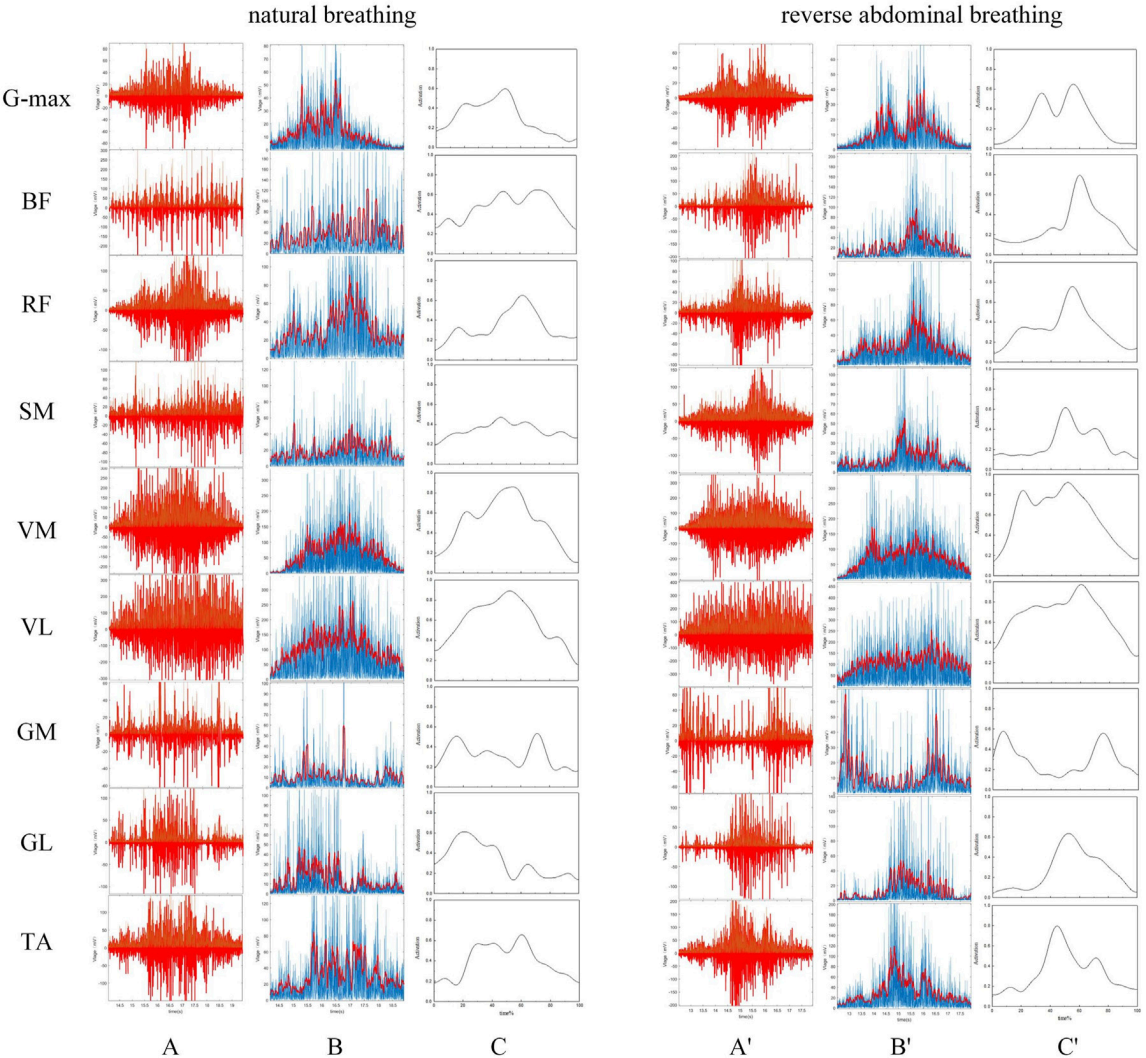
EMG Signal Processing. Panels **(A–C)** represent the filtering, rectification, and normalization of the EMG signals during natural breathing, while panels **(A′–C′)** represent the same processes during reverse abdominal breathing.

The raw EMG data were processed using a program written in MATLAB R2021a, which included filtering, rectification, and normalization. First, a fourth-order Butterworth bandpass filter with a frequency range of 20–500 Hz was applied to filter the signal (Date et al., 2021). To better display the trend of the EMG signal and reflect the temporal changes in muscle activation, a 2nd-order Butterworth high-pass filter with a cutoff frequency of 50 Hz was used to remove high-frequency noise interference. The data were then full-wave rectified, and the envelope was extracted and smoothed. Finally, the extracted envelope was normalized. The vertical axis was normalized by the maximum amplitude of each channel, resulting in a muscle activation level ranging from 0 to 1, representing no activation to full activation. The horizontal axis was normalized using linear interpolation, scaling the time axis to a range of 0%–100%. Through this standardized sequence of signal filtering, full-wave rectification, envelope extraction, and normalization, the EMG data are effectively denoised and individual variability is minimized, thereby providing a consistent foundation for subsequent analysis of muscle activation patterns across different breathing modes, as shown in Figure 2. The mean amplitude and root mean square (RMS) amplitude of the EMG signal were extracted for each participant, and the co-activation ratio was calculated, as shown in Equation 2.

$$Co-activation\;Ratio={RMS}_{Antagonist}/{RMS}_{Agonist}$$ (2)

### 2.5 Muscle synergy extraction

The EMG signals, after processing, were utilized to extract muscle synergies, which are constituted by the original EMG matrix M. We employed non-negative matrix factorization (NMF) to decompose the matrix M into a linear combination of muscle weights (W), representing the spatial structure of the coordination components, and activation coefficients (C), reflecting the time-varying activation of these components. The values of W indicate the contribution of each muscle to the coordination component, while the changes in C reflect the CNS’s activation of specific coordination components at different time points (Kim et al., 2018). The values of W and C range from 0 to 1. A W value close to 0 indicates a minimal contribution of the muscle to the coordination component, while a value close to 1 indicates a larger contribution. When W > 0.3, the muscle is considered to be a major contributor to the coordination component (Xiong, 2019). Similarly, a C value close to 0 indicates that the coordination component is nearly inactive, whereas a value close to 1 indicates that the component is highly activated. The NMF formula used in this study is expressed as follows:

$$M_{\left(m\times n\right)}=W_{\left(m\times k\right)}{\times C}_{\left(n\times k\right)}$$ (3)

In Equation 3, $M_{\left(m\times n\right)}$ represents the original EMG matrix with m EMG channels and n sampling points. $W_{\left(m\times k\right)}$ represents the muscle synergy weights for m muscles and k coordination components, while $C_{\left(n\times k\right)}$ represents the activation coefficients for n sampling points and k coordination components. In this study, m is set to 9, and n is standardized to 100, resulting in an original matrix M of size 9 × 100.

This study involves 9 muscles. To determine the minimum number of coordination components k, multiple iterations of the M matrix were computed and analyzed for values of k ranging from 1 to 9, yielding a reconstructed matrix M′. The final number of coordination components was determined based on the variance accounted for (VAF) between M and M′. The criterion for selecting the number of coordination components was that VAF>95% and the rate of change in VAF was less than 1% (Li et al., 2022). This standard ensured that the chosen muscle synergy components sufficiently reconstructed muscle activity under each condition.

$$VAF=1-{\left(M-M^{\prime }\right)}^{2}/M^{2}$$ (4)

In Equation 4′ represents the reconstructed matrix, and VAF denotes the variance accounted for between the original EMG matrix and the reconstructed matrix. The VAF value ranges from 0% to 100%, with higher values indicating better reconstruction quality. This value is used to assess the degree of reconstruction of the matrix M′ relative to the original matrix M.

**FIGURE 3:**
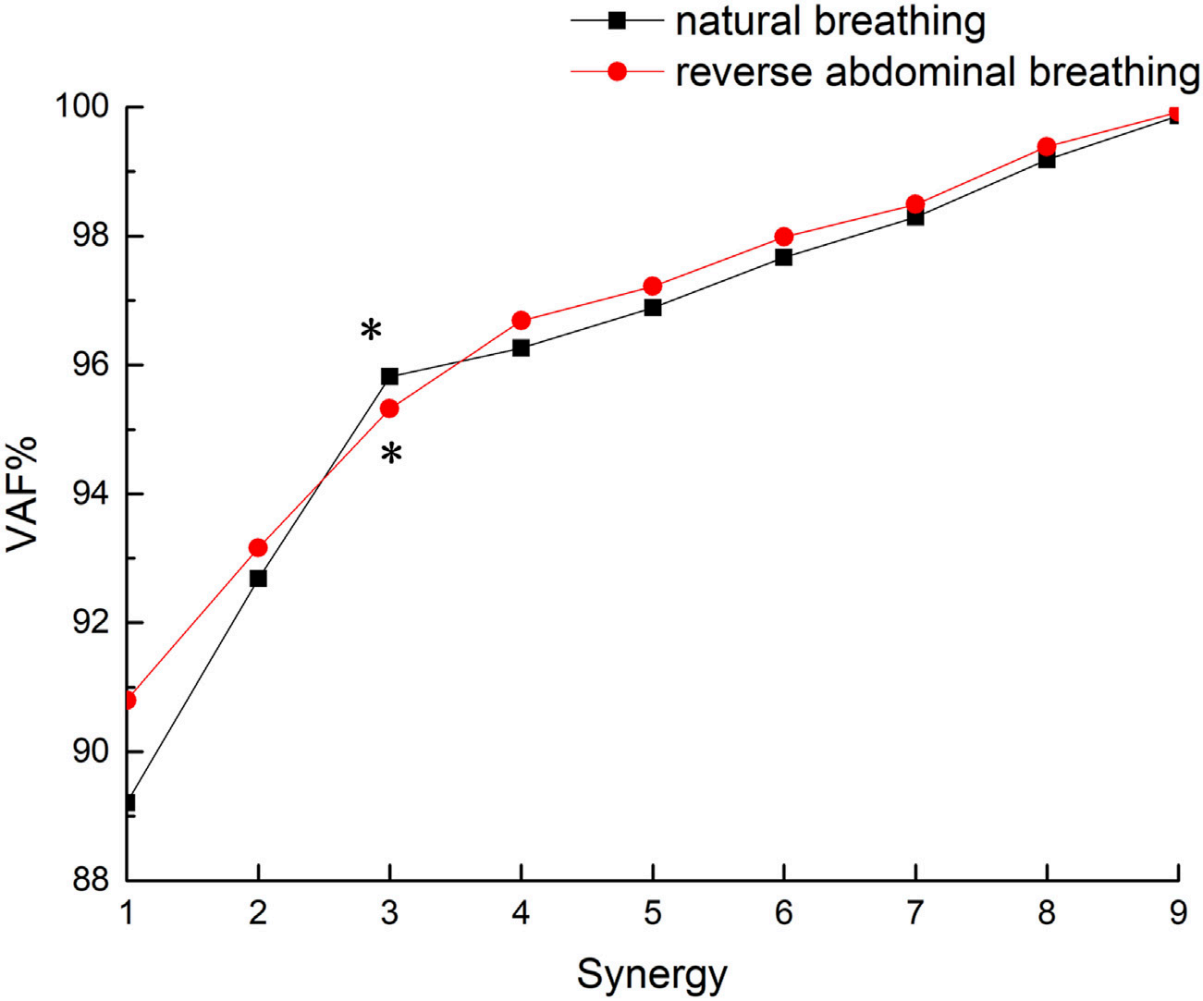
VAF values for the two breathing methods.

The VAF values for the two breathing conditions in this study are shown in Figure 3. Both breathing patterns met the criteria (VAF >95% with a rate of VAF change <1%) at k = 3 (natural breathing: VAF = 95.82% with a rate of VAF change 0.86%; reverse abdominal breathing: VAF = 95.32% with a rate of VAF change 0.72%), so the final number of muscle synergy components was determined to be 3.

**FIGURE 4:**
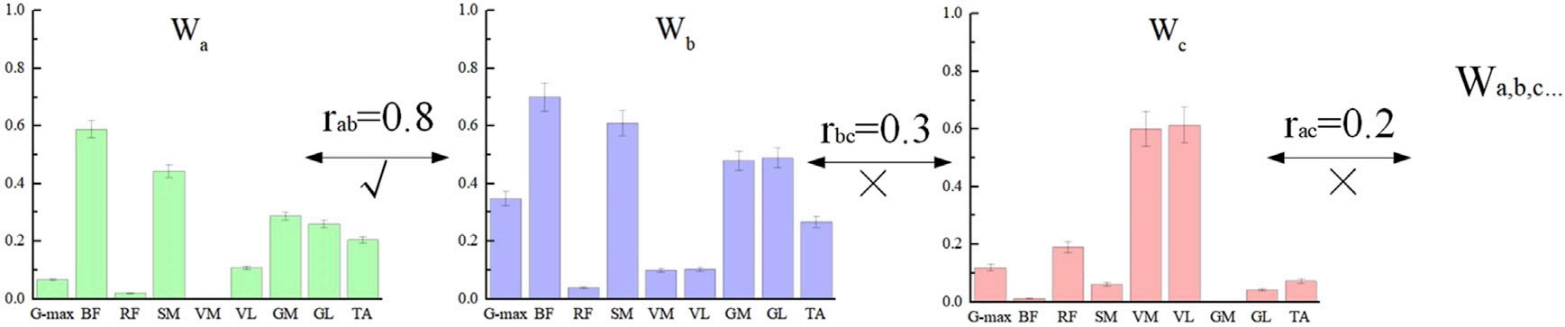
Muscle synergy weight classification. Labels a, b, and c represent three of the many subjects. Since the correlation coefficient between a and b is greater than 0.6, they are classified into the same group.

Finally, to obtain the overall characteristics of the muscle synergy patterns for different participants under the same breathing condition, we classified the muscle synergy weights W of all participants who completed NMF using the Pearson correlation coefficient r. First, the muscle synergy weights W from all participants were combined into a single matrix, and the similarity of the matrix vectors was assessed. When r > 0.6 (Yokoyama et al., 2016), the muscle synergy components were classified into the same group, as shown in Figure 4. Each W was assigned to only one group. If a W was similar to two or more muscle synergy components, it was classified into the group with the higher r value. After the classification of muscle weights W, the corresponding time activation curves C were automatically grouped into the same category (Yang, 2022). In this study, the final number of muscle synergy components under both breathing conditions was determined to be 3, which were named Synergy1-3, abbreviated as Syn1-3. These synergies reflect underlying functional modules involved in motor coordination during TCC movements. The same number of synergies indicate a similar level of motor control efficiency, while further analysis of synergy structure can help reveal how different breathing patterns influence muscle coordination strategies.

### 2.6 Statistical methods

Statistical analysis was performed using SPSS 21.0. The normality of the parameters was assessed using the Shapiro-Wilk test. For data that followed a normal distribution, paired sample t-tests were used, while for data that did not follow a normal distribution, the Wilcoxon signed-rank test was applied. A significance level of p = 0.05 was set, and data are presented as mean ± standard deviation (M ± SD).

## 3 Results

### 3.1 Model validation

**FIGURE 5:**
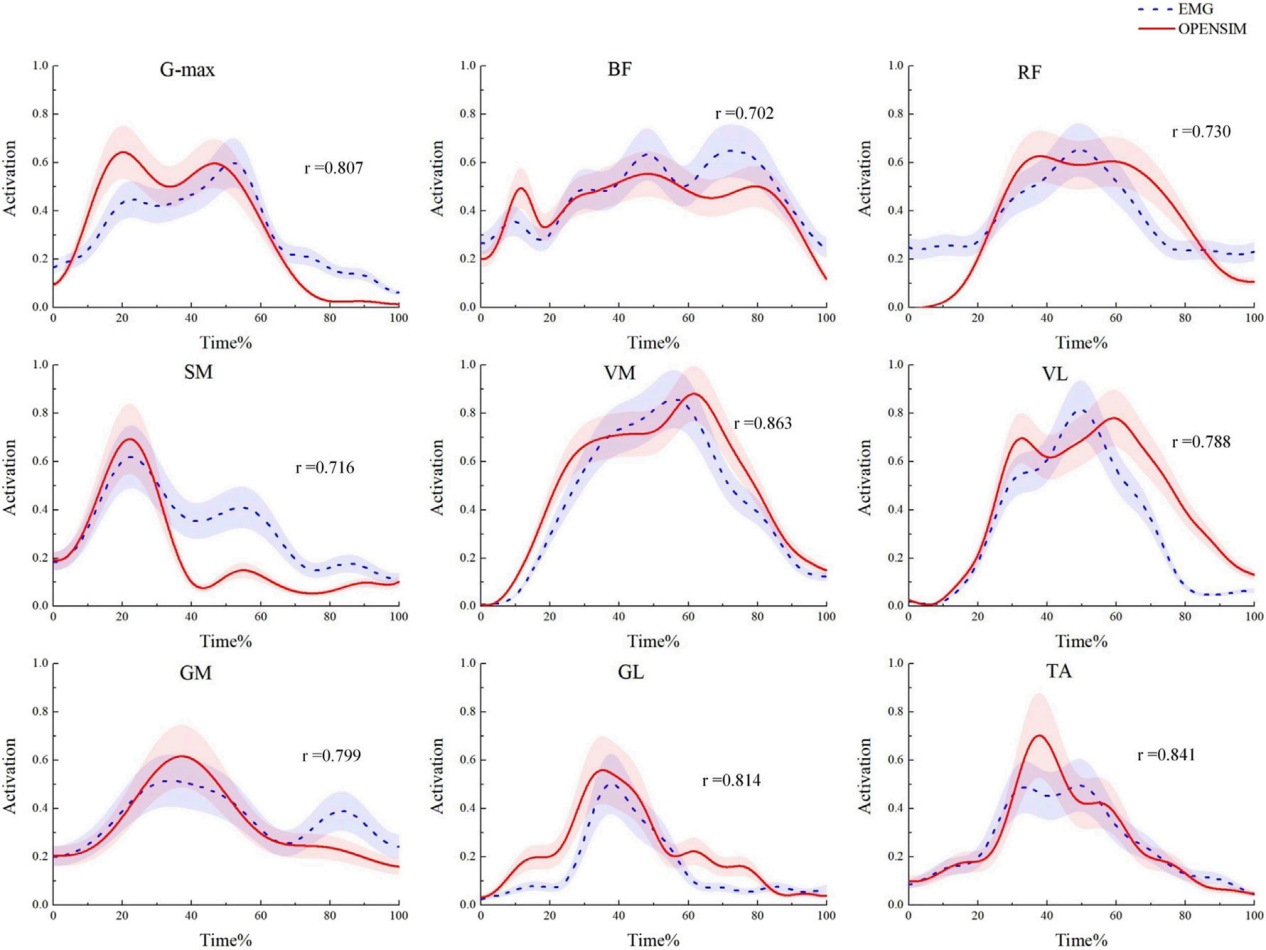
Model validation diagram. Comparison of muscle activation levels obtained from EMG signals and the OpenSim static optimization algorithm.

During the model validation process, in order to assess the accuracy and validity of the simulation model, the EMG envelope extracted from experimental records was compared with the muscle activation values obtained through static optimization in OpenSim. The results are shown in Figure 5. The average Pearson correlation coefficient for nine muscles was 0.784, indicating a strong positive correlation between the two, suggesting that the OpenSim simulation data in this study are reliable (Hua et al., 2024).

### 3.2 Peak muscle force, joint stiffness, and muscle co-activation ratio under two breathing modes

As shown in Table 1, by comparing the muscle forces calculated through static optimization in OpenSim, we found that the peak muscle force of the G-max during the TCC reverse abdominal breathing movement was significantly greater than that during natural breathing (P = 0.024). The peak muscle force of the RF was significantly greater than that during natural breathing (P = 0.036), the peak muscle force of the SM was significantly greater than that during natural breathing (P = 0.039), and the peak muscle force of the TA was significantly greater than that during natural breathing (P = 0.026). The peak muscle force of the BF was notably greater than that during natural breathing (P = 0.008). The two breathing methods also had different effects on lower limb joint stiffness. The knee joint stiffness under reverse abdominal breathing was significantly greater than that under natural breathing (P = 0.037), and the ankle joint stiffness was significantly greater than that under natural breathing (P = 0.029). Regarding muscle co-activation ratios, the co-activation ratio of the GL to the TA during TCC reverse abdominal breathing was significantly lower than that during natural breathing (P = 0.034).

**TABLE 1:** Index parameters of two breathing modes in TCC supporting gait movements.

Parameters	Values	Natural breathing	Reverse abdominal breathing
Peak Muscle Force (N/BW)	G-max	0.513 ± 0.084	0.794 ± 0.099^*^
	BF	1.079 ± 0.054	1.472 ± 0.008^**^
	RF	1.627 ± 0.089	1.873 ± 0.112^*^
	SM	0.437 ± 0.064	0.569 ± 0.082^*^
	VM	1.782 ± 0.065	1.807 ± 0.062
	VL	2.556 ± 0.069	2.599 ± 0.071
	GM	1.898 ± 0.053	1.911 ± 0.075
	GL	2.351 ± 0.075	2.358 ± 0.064
	TA	0.857 ± 0.006	1.139 ± 0.007^*^
Stiffness (Nm/kg/°)	Hip Joint	−0.011 ± 0.003	−0.009 ± 0.004
	Knee Joint	−0.063 ± 0.058	−0.097 ± 0.043^*^
	Ankle Joint	−0.009 ± 0.039	−0.036 ± 0.021^*^
Co-activation Ratio	RMS_(BF+SM)_/RMS_(RF+VM+VL)_	0.424 ± 0.093	0.427 ± 0.022
	RMS_TA_/RMS_GL_	1.543 ± 0.075	1.776 ± 0.116^*^

*Indicates: P < 0.05, ** Indicates: P < 0.01.

### 3.3 Muscle synergy under two breathing modes

**FIGURE 6:**
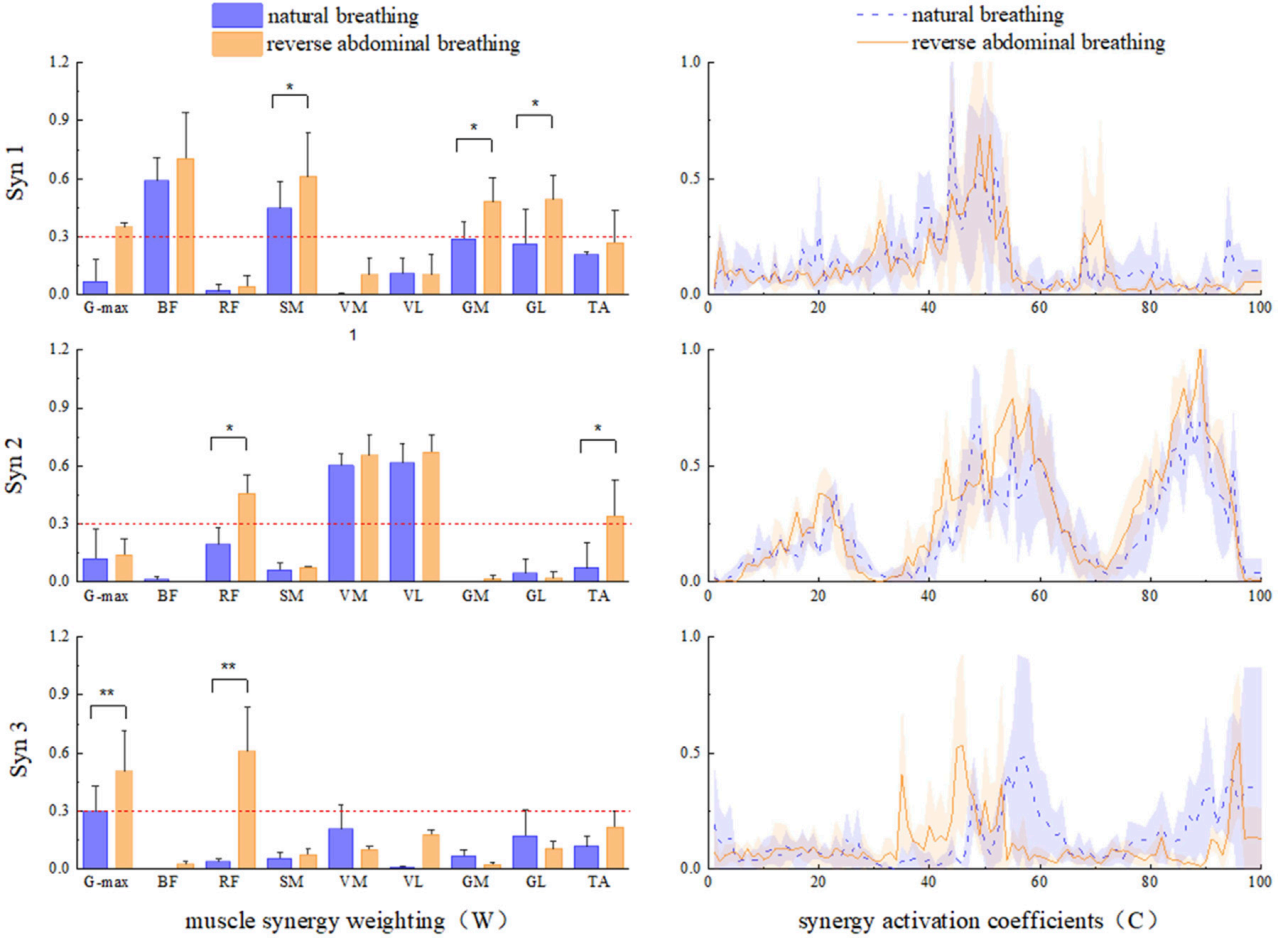
The muscle synergy pattern of two breathing modes in TCC supporting gait movements. *Indicates: P < 0.05, ** Indicates: P < 0.01.

The three muscle synergy patterns for the two breathing methods are shown in Figure 6. In Syn1, during reverse abdominal breathing, the relative weight of the SM was significantly higher than that during natural breathing (P = 0.35), the relative weight of the GM was significantly higher than that during natural breathing (P = 0.39), and the relative weight of the GL was significantly higher than that during natural breathing (P = 0.28). In Syn2, during reverse abdominal breathing, the relative weight of the RF was significantly higher than that during natural breathing (P = 0.23), and the relative weight of the TA was significantly higher than that during natural breathing (P = 0.26). In Syn3, during reverse abdominal breathing, the relative weight of the G-max was highly significantly higher than that during natural breathing (P = 0.009), and the relative weight of the RF was highly significantly higher than that during natural breathing (P = 0.002). The co-activation coefficient curves for Syn1 and Syn2 showed no significant differences, whereas the peak activation of Syn3 during reverse abdominal breathing occurred earlier than during natural breathing. The primary muscles involved in Syn3 were the G-max and RF, indicating that reverse abdominal breathing activates these two muscles earlier than natural breathing.

## 4 Discussion

### 4.1 Muscle activation and joint stiffness characteristics analysis under two breathing modes

The results of this study support the hypothesis that TCC reverse abdominal breathing enhances lower limb muscle activation. Specifically, compared to natural breathing, TCC reverse abdominal breathing significantly increases the muscle strength of the G-max, RF, BF, SM, and TA, as well as the stiffness of the knee and ankle joints and the co-activation ratio in the calf muscles.

Reverse abdominal breathing in TCC refers to contracting the abdomen during inhalation, which draws the breath downward and stabilizes the core. This technique differs from natural breathing and helps enhance intra-abdominal pressure. To ensure “posture-breathing synchronization,” the postural control system must account for the continuous disturbance of the body’s center of mass. Posture selection and transitions between patterns depend on the degree of stability and the movement strategy pre-selected based on the task at hand (Bardy et al., 1999). In yoga practice, the active contraction of the abdomen during inhalation is similar to the characteristics of reverse abdominal breathing, which helps to enhance the strength and flexibility of the diaphragm and promotes deeper breathing. Kelley et al. (2019) studied yoga postures and found that in yoga, the average EMG amplitude of the TA and gastrocnemius in the ankle joint was significantly higher than that of the BF and RF in the thigh, reflecting the dominance of ankle joint muscles in single-leg support. This suggests that in yoga postures, muscle activation is primarily used to regulate postural balance via the ankle joint strategy. A previous postural sway model (Creath et al., 2005) found that when the body begins to experience minor disturbances, the ankle joint strategy is activated first to adjust the body’s movement and return the center of mass to a stable position. Since trunk rotation in an upright posture is dominated by ankle plantarflexion, and both the trunk and leg move in the same direction, postural responses after disturbances can be explained by a trade-off between energy expenditure and postural stability. In cases where activation of hip joint muscles is unnecessary, adjusting the ankle joint to minimize mechanical work can effectively shift the center of mass to stability (Afschrift et al., 2016). Given that the TCC natural breathing condition showed low calf muscle co-activation but relatively high RMS EMG of the gastrocnemius, TCC natural breathing may adjust the ankle joint strategy as a basis for posture control.

The disturbance of the support base by reverse abdominal breathing leads to moderate adjustments in postural muscle activity and body positioning. This may be due to the increase in intra-abdominal pressure and deep core stability, which alters muscle recruitment patterns in the lower limbs. A possible explanation is that TCC reverse abdominal inhalation causes an elevation of the pelvic floor and expansion of the posterior diaphragm, resulting in increased activation of the G-max, RF, and hamstrings, which in turn helps to smoothly transition the center of mass from the hips to the feet. Considering that TCC reverse abdominal breathing is associated with the recruitment of hip muscles around the lower limb during knee flexion tasks, the diaphragm’s co-activation with the core muscles adjusts lower limb muscle activity to control body movement and maintain a stable posture. This ensures that the center of mass remains within the support base (Winter et al., 1998), increasing hip joint stability while reducing ankle joint motion amplitude. Moreover, in this study, the activation of the GM during TCC reverse abdominal breathing was not significantly higher than that during natural breathing, while the BF and RF both exhibited higher activation levels during the support phase compared to natural breathing. Therefore, from the perspective of posture adjustment, this reverse abdominal breathing technique in TCC increases the activation levels of the G-max, RF, and SM around the hip joint.

The co-activation of agonist and antagonist muscles participates in the regulation of joint stiffness and postural instability (Hortobagyi and DeVita, 2000; Schmitz et al., 2009). A higher level of co-activation may affect the ability of the knee extensors to generate force over short periods (Arampatzis et al., 2007). On one hand, passive stiffness at the ankle joint reflects the relationship between body balance, movement performance, and net joint torque (Arampatzis et al., 2005). Simultaneously, the coordinated activity of all muscles crossing the joint largely determines the net torque and joint stiffness, thereby fulfilling the demands for body balance and movement performance (Cop et al., 2021; Nagamori et al., 2016). Therefore, changes in breathing patterns that increase muscle co-activation—especially in the lower legs—may directly contribute to increased stiffness for enhanced stability. This suggests that in this study, reverse abdominal breathing TCC led to significantly higher ankle joint stiffness compared to natural breathing. Through ankle strength exercises in strength and endurance training, better development of passive ankle stiffness can be achieved. On the other hand, excessively high stiffness is considered to increase musculoskeletal load, thereby raising the risk of injury, while excessively low stiffness is thought to allow excessive joint movement, thus increasing the risk of soft tissue damage (Butler et al., 2003). Previous studies have suggested that by adopting strategies to reduce the proportion of knee joint torque usage and increase the proportion of hip joint torque usage, the total available torque can be redistributed (Parijat and Lockhart, 2008; Hortobágyi et al., 2003). However, the results of this study indicate that TCC lower limb stiffness is much lower than that of high-intensity movements, and the small to moderate stiffness changes brought by TCC gait training are unlikely to increase the risk of injury during exercise (Davis and Gruber, 2021).

Currently, it is unclear how the reverse abdominal breathing method specifically affects lower limb stiffness in TCC, as reverse abdominal breathing does not influence hip joint stiffness. One explanation could be due to an increase in co-activation ratios of the lower leg muscles, while another explanation could be related to the anatomical positioning of the RF and BF. The RF originates from the anterior inferior iliac spine and the superior rim of the acetabulum, while the long head of the BF originates from the ischial tuberosity. These muscles, crossing the hip joint, contribute to maintaining hip posture stability, which may increase the system’s passive stiffness. However, the study did find that reverse abdominal breathing significantly increased knee and ankle joint stiffness compared to natural breathing. Furthermore, considering the observed correlation between stiffness and muscle co-activation, reverse abdominal breathing helps maintain body balance and minimize postural instability. Given that our findings reflect higher muscle force of the RF and BF during the shift of the center of gravity with reverse abdominal breathing, the eccentric control of knee and hip joint stability is likely enhanced. This aligns with previous studies, as muscle force effectiveness depends on different forms of movement. Breathing increases the hamstring recruitment ability associated with the level of quadriceps activation during squatting (Paillard, 2017). This likely requires balance responses through the center of pressure (COP) trajectory, with knee extensor eccentric actions controlling knee flexion during squatting (Voukelatos et al., 2007; Pai et al., 2000), as improved stiffness at initial heel contact can better stabilize joints against unforeseen disturbances. Due to the characteristics of reverse abdominal breathing in TCC, emphasizing the rotation of the torso and synchronized diaphragmatic breathing, diaphragmatic breathing can increase muscle activation and proprioception (D'Amiri and Zemková, 2023). Enhanced core muscle strength and proprioceptive function may contribute to improved postural stability, and diaphragmatic breathing patterns may also be considered in relation to improving posture control. Given the minimization of mechanical work and postural instability, movement strategies can be chosen accordingly, such as minimizing mechanical work at the ankle joint or minimizing postural instability at the hip joint (Afschrift et al., 2016). Therefore, it is suggested that reverse abdominal breathing in TCC may be influenced by a combination of active or passive mechanisms affecting lower limb muscle force and joint stiffness.

### 4.2 Muscle synergy analysis under two breathing modes

This study compared the neuromuscular responses of lower limb muscle groups during natural breathing and reverse abdominal breathing in TCC. The main findings indicate that Syn1 W1 was primarily activated in the BF, SM, GM, GL, and TA during both natural and reverse abdominal breathing. However, during reverse abdominal breathing, the relative weight of SM, GL, and GL was significantly higher compared to natural breathing, consistent with the ankle strategy-hip strategy. In Syn2 W2, both natural and reverse abdominal breathing mainly activated the VM, VL, RF, and TA, with the relative weight of RF and TA significantly higher during reverse abdominal breathing, consistent with the knee strategy. In Syn3 W3, both natural and reverse abdominal breathing activated the G-max and RF, with the relative weight of G-max and RF significantly higher during reverse abdominal breathing compared to natural breathing, consistent with the hip strategy. We also observed that in W2 and W3, postural perturbations reduced the muscle weight of knee flexors, specifically the BF and SM (below 0.3).

It has been reported that voluntary breathing can activate cortical areas, including the primary motor cortex (M1) associated with respiratory muscles, the premotor area, and the supplementary motor area (Petersen et al., 2011). Both respiratory and limb muscles can be controlled by M1, so different forms of breathing regulation depend on central commands sent from M1 to the respiratory muscles (Stendardi et al., 2005). One study evaluated the cortical spinal excitability of the VL during isometric contractions with different breathing techniques using surface EMG recordings of motor evoked potentials during transcranial magnetic stimulation. It found that motor evoked potentials were significantly higher during purposeful inhalation and exhalation compared to normal breathing (Cleland et al., 2023). The activation of afferent signals from the respiratory system and the interaction with motor cortex excitability suggest that limb movement is related to purposeful breathing control. During voluntary breathing, cortical spinal excitability of finger muscles increases, indicating that breathing and the related cortical motor areas enhance motor function, and activating these motor areas related to breathing may improve the motor function of non-respiratory muscles (lower limb muscles) (Li and Rymer, 2011; Shirakawa et al., 2015). Given that the abdominal muscles (rectus abdominis, diaphragm, and internal/external obliques) are primarily involved in reverse abdominal breathing in TCC, it is possible that the activity of the CNS M1 will activate abdominal muscles and their associated lower limb muscles in certain synergy components (W1, W2, W3). During TCC reverse abdominal breathing, abdominal muscles are first activated, followed by the contraction of the diaphragm and internal/external obliques, and then the diaphragm is contracted again, with the pelvic floor muscles activated last. Considering that the nervous system controls a small number of functional synergies through dimensional reduction rather than individually controlling each muscle activation (Ghislieri et al., 2020), this reflects a group of muscles activated at a fixed ratio over a certain period or synergistically activated at different times. Therefore, the rectus abdominis and internal/external obliques are in the same muscle synergy component W1 within CNS regulation, leading to high activation levels of the BF, SM, GM, GL, and TA in Syn1 (W1); the diaphragm, VM, VL, RF, and TA are in the same muscle synergy component W2, leading to significantly higher activation levels of the RF and TA; the pelvic floor muscles, G-max, and RF are in the same muscle synergy component W3, leading to high activation levels of the G-max and RF. Meanwhile, reverse abdominal breathing in TCC requires deep and prolonged breathing coordinated with movements, with abdominal muscles (rectus abdominis, internal/external obliques), diaphragm, and pelvic floor muscles working together. This coordination during the task enhances the synergy mechanism of respiratory muscles (abdominal muscles, diaphragm, pelvic floor muscles) and lower limb active muscles (Emerich Gordon and Reed, 2020), specifically the synergistic action of rectus abdominis, internal/external obliques with the lower limb muscles (BF, SM, GM, GL, TA), the diaphragm with the RF, and the pelvic floor muscles with the G-max and RF, resulting in higher activation levels. Therefore, reverse abdominal breathing in TCC recruits more lower limb muscles during inhalation, further supporting the hypothesis that TCC reverse abdominal breathing enhances lower limb muscle synergy.

On the other hand, the overall stability of the core region is composed of the respiratory muscle group formed by the diaphragm, abdominal muscles, pelvic floor muscles, and spinal extensors. The upward and downward movement amplitude of the diaphragm increases during the reverse abdominal breathing in TCC, while the abdominal muscles and pelvic floor muscles contract synergistically. The coordination between breathing rhythm and movement patterns generates greater intra-thoracic negative pressure (Talasz et al., 2022). This increases abdominal pressure through inhalation to activate the pelvic floor and abdominal wall muscles, thereby improving lower limb stability and the stability of the posterior-lateral and anterior-lateral regions. Given that TCC’s balance in both static (end of posture phase) and dynamic (turning) movements primarily focuses on stabilizing the core region of the waist and hips (Ferraro et al., 2020), it generates continuous potential postural stability. The proximal body parts are fixed, while the distal parts generate force, and the force is transmitted through muscle-tendon-bone chains. This results in the coordination of agonist, synergist, and antagonist muscles, forming either spatial (a group of muscles activated in a fixed ratio) or temporal (a group of muscles activated variably) coordination (Overduin et al., 2015). The muscle activation patterns exhibit significantly different spatiotemporal characteristics, indicating that different breathing methods in TCC, regulated by the CNS, trigger the synergistic activation of independent muscles. This activation occurs by reducing the dimensionality to control the functional synergy of a few muscles rather than controlling each muscle individually (Ghislieri et al., 2020). This shows different coordinated breathing muscle actions generating different “ankle strategies” or “knee strategies,” maintaining postural stability in the body. Furthermore, muscle synergy can occur by activating certain CNS, representing customized patterns that control specific task-level variables. These synergies can also be simultaneously activated, resulting in continuous potential postural responses that represent a mix of two different strategies (Torres-Oviedo and Ting, 2007). The various breathing muscle synergies generate different “ankle strategies” and “knee strategies” to maintain postural coordination. Therefore, this study found that reverse abdominal breathing Syn2 W2 produces a consistent “knee strategy,” while W3 generates a consistent “hip strategy” to maintain body stability. In contrast, reverse abdominal breathing Syn1 W1 mainly activates the hamstring (SM), gastrocnemius, and the “ankle strategy-hip strategy” consistently to maintain body coordination. As there is an inverse relationship between the synergistic effects produced by the muscle synergy components, meaning that the more the ankle strategy is activated, the less the knee and hip strategies are activated (Horak, 2006), it is inferred that the “hip strategy” of W3 and the “knee strategy” of W2 may influence the CNS’s regulation of posture strategies (one strategy increases while the other decreases) through breathing methods. Specific breathing methods may optimize the activation patterns of non-respiratory muscles (lower limb muscles) and influence the potential impact on movement-driven stability and coordination. Thus, the beneficial effects of practicing TCC may partly stem from the optimization and synergy of this multi-system interaction. In particular, it plays a significant role in improving the stability and coordination of the posture of the elderly.

## 5 Limitations

This study has several limitations that should be acknowledged. First, the small sample size may limit the statistical power and generalizability of the findings; future research should aim to recruit a larger and more diverse cohort. Second, the absence of a control group makes it difficult to isolate the specific effects of the breathing techniques on muscle activation. Additionally, the study focused on a single TCC movement, which may not represent the full range of TCC practice; examining multiple movements could enhance generalizability. Moreover, only two breathing techniques were analyzed; future investigations should consider including other common TCC breathing methods to provide a more comprehensive understanding. Finally, future studies could integrate a more comprehensive analysis of dynamic parameters—such as joint kinetics, muscle force generation, and joint stability—together with advanced technologies like wearable EMG sensors or real-time muscle force measurement, to enhance both the understanding of breathing technique effects and the precision and applicability of the findings.

## 6 Conclusion

Reverse abdominal breathing led to greater activation of key muscles such as G-max, RF, and SM, which are crucial for stability and postural control during TCC movements. In terms of muscle synergy, the natural breathing and reverse abdominal breathing W1 primarily activates the BF, SM, GM, GL, and TA. However, the relative activation of the SM, GM, and GL is significantly higher during reverse abdominal breathing compared to natural breathing, consistent with the ankle strategy-hip strategy a postural control mechanism that involves coordinated use of both the ankle and hip joints to maintain balance and stability during TCC movements. W2 with natural breathing and reverse abdominal breathing mainly activates the VM, VL, RF, and TA, with the relative activation of the RF and TA during reverse abdominal breathing significantly higher than during natural breathing, in alignment with the knee strategy. W3 activates the G-max and RF in both natural and reverse abdominal breathing, consistent with the hip strategy to enhance postural stability. The specific breathing methods have optimized activation patterns that influence non-respiratory muscle movement, and the synchronization of stability and coordination in motor behavior is executed through CNS feedback. These findings suggest that reverse abdominal breathing could be particularly effective for improving postural stability and coordination in individuals with over 5 years of TCC experience, making it a valuable tool for both TCC practice and rehabilitation programs. While these findings are specific to the ‘Wild horse parts its mane’ movement, further research is needed to explore whether these results hold true across other TCC movements and breathing techniques.

## Data Availability

The raw data supporting the conclusions of this article will be made available by the authors, without undue reservation.
